# Intraoperative severe gastric venous congestion during total pancreatectomy with replaced common hepatic artery: a case report

**DOI:** 10.1186/s40792-024-01983-x

**Published:** 2024-08-08

**Authors:** Takahiro Yamanaka, Kenichiro Araki, Hideki Suzuki, Hidenobu Osawa, Ken Shirabe

**Affiliations:** 1 Department of General Surgical Science, Division of Hepatobiliary and Pancreatic Surgery, Graduate School of Medicine, Gunma University, 3-39-22 Showamachi, Maebashi, Gunma 371-8511 Japan; 2 Department of Surgery, Isesaki Municipal Hospital, Gunma, Japan

**Keywords:** Total pancreatectomy (TP), Gastric venous congestion (GVC), Replaced common hepatic artery (RCHA)

## Abstract

**Background:**

Gastric venous congestion (GVC) is one of the complications of total pancreatectomy (TP). Here, we report a case of intraoperative severe GVC during TP with a replaced common hepatic artery (RCHA).

**Case presentation:**

A 65-year-old female patient was diagnosed with intraductal papillary mucinous carcinoma. Her CHA branched from the superior mesenteric artery as RCHA. She underwent subtotal stomach preserving TP. The tumor was resected with splenic artery (SpA) and total gastric vein transections. Severe GVC and bleeding from the stomach tube occurred intraoperatively. A strong pulsation was observed in the left gastric artery (LGA), and we suspected an increased blood flow from the celiac artery (CeA) to the LGA after SpA resection. Total gastrectomy (TG) was then performed to control the severe GVC-related bleeding. The patient was discharged without complications 19 days postoperatively.

**Conclusion:**

TP with RCHA may increase the risk of severe GVC due to increased blood flow from CeA to LGA.

## Background

Total pancreatectomy (TP) is a surgical procedure for patients with pancreatic diseases [1]. TP results in gastric venous congestion (GVC) at 5.3–27.9% of cases [2] [3] [4]. Additionally, GVC with TP demonstrated a high mortality rate (7.4%) [4]. We report a case of intraoperative severe GVC during TP with a replaced common hepatic artery (RCHA).

## Case presentation

**Fig. 1:**
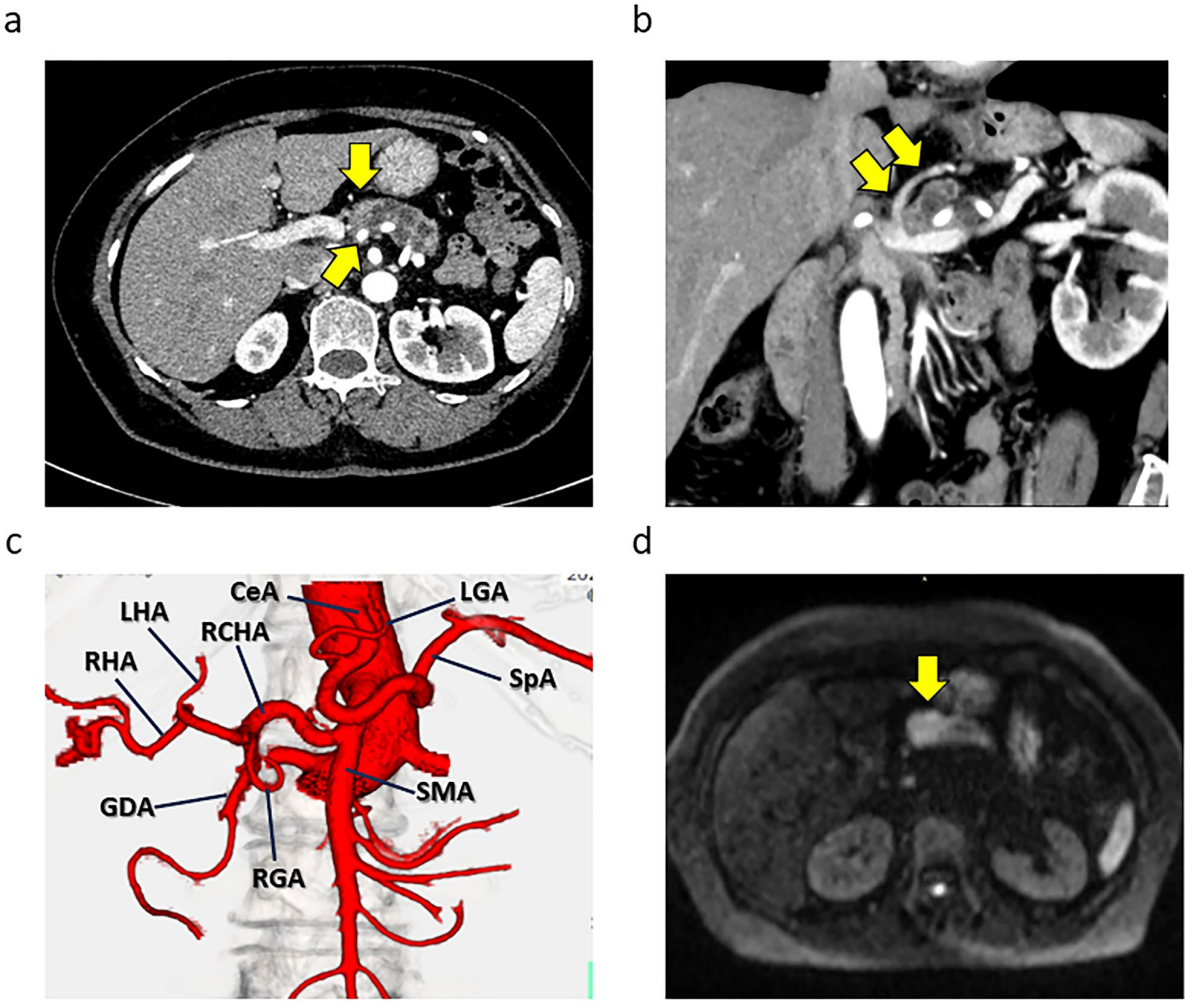
Computed tomography (CT) and magnetic resonance imaging (MRI) of the case. **a** The enhanced CT detected the pancreatic body tumor and main pancreatic duct dilation in the pancreatic body and tail. The arrows indicated that the tumor was in contact with splenic artery (SpA). **b** The enhanced CT shows that the tumor was in contact with the left gastric vein (LGV). The arrows indicated the LGV. **c** Three-dimensional artery image from enhanced CT. Celiac artery (CeA), left gastric artery (LGA), SpA, superior mesenteric artery (SMA), replaced common hepatic artery (RCHA), left hepatic artery (LHA), right hepatic artery (RHA), gastroduodenal artery (GDA) and right gastric artery (RGA) were indicated. **d** MRI diffusion-weighted image revealed high intensity in the pancreatic body. The arrow indicated the tumor

**Fig. 2:**
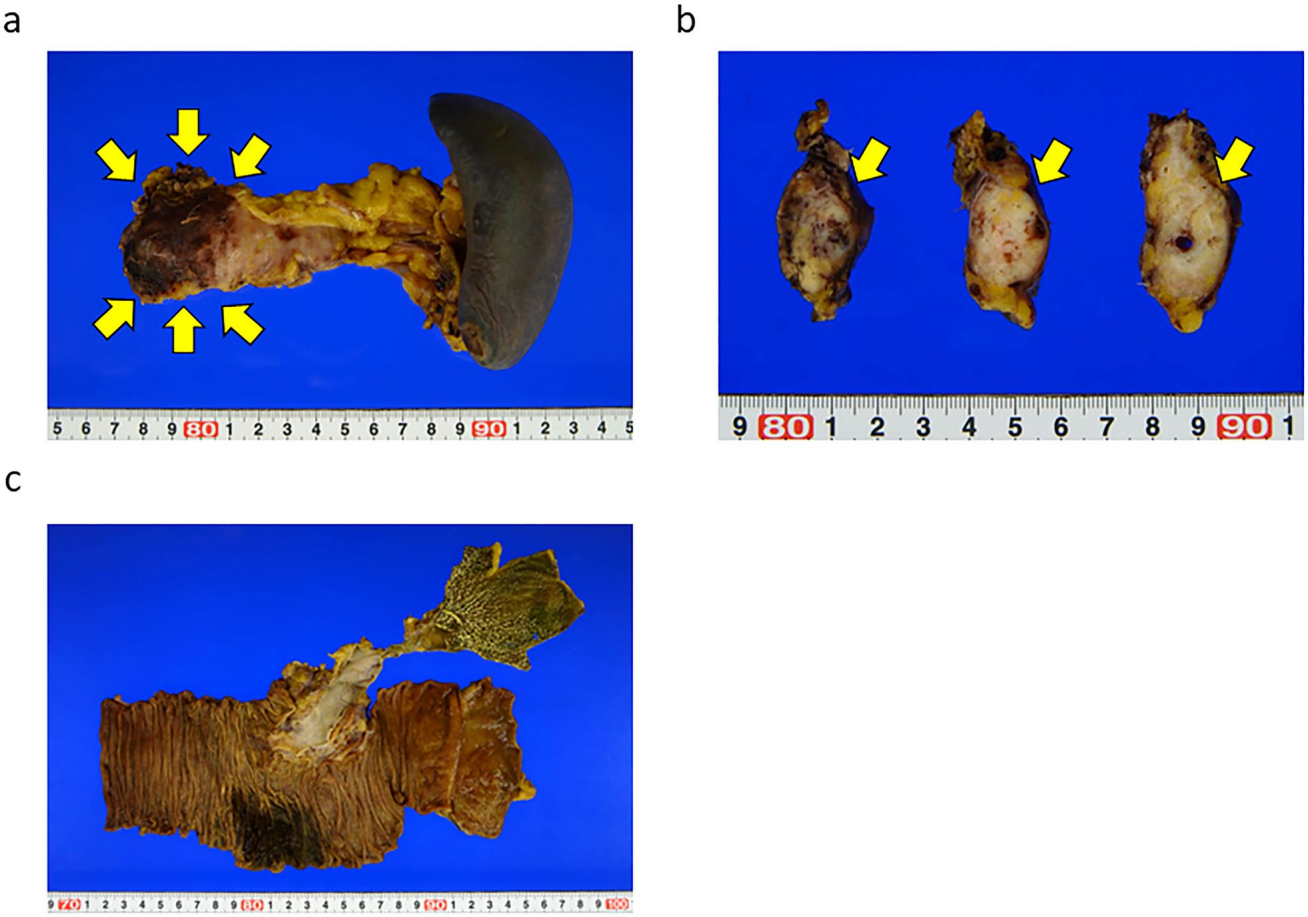
The images of resection specimens of the case. **a**, **b** Distal pancreatectomy (DP) was performed and the tumor was detected in the pancreatic body. The intraoperative frozen section diagnosis revealed residual cancer at the pancreatic stump. The arrows indicated the tumor. **c** The subtotal stomach preserving TP (SSPTP) was performed (pancreaticoduodenectomy was performed adding to DP)

A 65-year-old female patient with no medical history had been observed for intraductal papillary mucinous neoplasia (IPMN) with main pancreatic duct dilation in the pancreatic body and tail at the local hospital for a few years. She was suspected with a malignant pancreatic tumor and visited our hospital. Laboratory data on hospital admission included white blood cell counts of 6500/μl, red blood cell count of 506 × 104/μl, hemoglobin of 13.7 g/dl, hematocrit of 42.1%, platelets of 25.4 × 104/μl, total protein of 7.4 g/dl, albumin of 4.4 g/dl; total bilirubin of 0.77 mg/dl, aspartate aminotransferase of 26 IU/l, alanine aminotransferase of 25 IU/l, alkaline phosphatase of 68 IU/l, γ-glutamyl transferase of 20 IU/l, amylase of 15 U/l, creatine kinase of 44 IU/l, lactate dehydrogenase of 278 IU/l, blood urea nitrogen of 14 mg/dl, creatinine of 0.65 mg/dl, Na of 140 mEq/dl, K of 4.3 mEq/dl, Cl of 103 mEq/dl, C-reactive protein of 0.25 mg/d, carcinoembryonic antigen of 1.7 ng/ml, CA19-9 of 109.1 U/ml, DUPAN-2 of 79 U/ml, and SPAN-1 of 17 U/ml. Computed tomography (CT) detected a mass of approximately 3 cm in the pancreatic body, dilation of the main pancreatic duct, and a cystic structure suspected of IPMN in the pancreatic tail. The tumor was in contact with the splenic artery (SpA) and left gastric vein (LGV) but was not in contact with the superior mesenteric artery (SMA) or celiac artery (CeA) (shown in Fig. 1a, b). An evaluation of the arteries based on three-dimensional pictures revealed the RCHA originating from the SMA (shown in Fig. 1c). Magnetic resonance imaging revealed a high-intensity signal in the mass of the pancreatic body with a diffusion-weighted image (shown in Fig. 1d). Endoscopic ultrasound-guided fine needle aspiration detected adenocarcinoma. The patient was then diagnosed with intraductal papillary mucinous carcinoma cT2N0M0StageIB (UICC 8th) and planned to undergo surgery. Pancreaticoduodenectomy was not appropriate as a curative procedure because the tumor was located in the body of the pancreas and was also suspected to be invading the SpA. So, we decided to perform a distal pancreatectomy (DP) with the SpA dissection. In the surgical findings, the tumor was contact with the SpA and the LGV. Intraoperative ultrasound was used to confirm the extent of the tumor and right gastroepiploic vein (RGEV) was transected because it closed to the right edge of the tumor. DP was performed with resecting the pancreas at the left end of the gastroduodenal artery (GDA). The intraoperative frozen section diagnosis indicated residual cancer at the pancreatic stump of DP, and we decided to perform the subtotal stomach preserving TP (SSPTP) because additional pancreatic resection was deemed impossible. The right gastric vein (RGV) was determined to be very thin and ineffective as a drainage vein and therefore could not be preserved when performing SSPTP. In summary, SpA, LGV, left gastroepiploic artery, left gastroepiploic vein, short gastric artery, short gastric vein, and RGEV were transected during the DP, and RGV, right gastric artery and right gastroepiploic artery were transected during the change to SSPTP, so no gastric drainage veins could be preserved and only the left gastric artery (LGA) supplied blood flow to the stomach. Immediately after the specimen has been extracted, a GVC and massive bleeding from the nasogastric tube occurred. Strong pulsation in the LGA was observed, and we suspected increased blood flow in the LGA after the resection the SpA. Gastric vein reconstructions were technically impossible; thus, a partial gastrectomy was performed to remove the particularly congested pyloric region of the remnant stomach. However, the GVC and bleeding demonstrated no improvement. We determined the necessity of LGA dissection to control the bleeding with the severe GVC and performed total gastrectomy (TG) to avoid postoperative gastric necrosis. The patient was discharged from the hospital 19 days postoperatively without complications. Pathological diagnosis included pancreatic adenocarcinoma, wel > por > mod, Pb, TS2(25 mm), infiltrative type, sci, INFc, ly1, v1, ne3, mpd0, pCH0, pDU0, pS1, pRP1, pPV0, pA0, pPLX, pOO0, pBCM0, pDPM1, R1, pN1(3/27), M0, pT2N1M0StageIIB (UICC 8th) (shown in Fig. 2a, b, c). Adjuvant chemotherapy was not administered due to her intension and physical condition. The patient had a local recurrence around the superior mesenteric vein and SMA 3 months postoperatively and started chemotherapy with gemcitabine plus nab-paclitaxel. Additionally, she changed the chemotherapy to FOLFIRINOX because of peritoneal dissemination 11 months postoperatively. She then currently continues her treatment 18 months postoperatively. She also received dietary therapy, insulin treatment, and lipase supplementation of the pancrelipase postoperatively, with serum albumin levels of 3.0 g/dl, 3.5 g/dl, and 3.0 g/dl, respectively, at 6, 12, and 18 months postoperatively.

## Discussion

In this case, RCHA branches off from SMA. A variant origin of CHA arising from either SMA or the aorta was reported in 4.0% of patients [5]. In cases with RCHA, the CeA branches only into the SpA and LGA. Resuscitative endovascular balloon occlusion of the aorta generally increases the arterial blood flow in patients with traumatic shock by blocking peripheral arteries [6]. It was also reported that an embolization of SpA had increased hepatic arterial blood flow in patients with splenic hepatic artery steal syndrome [7] [8]. Thus, SpA transection should significantly increase the blood flow from the CeA to the LGA in the case with RCHA. The increased blood flow of the LGA may then cause severe GVC.

Gastrectomy was one of the procedures to improve GVC after TP. It was reported that gastrectomies (sub-TG: 10.7% and TG: 0.7%) had been performed with TP, and LGV dissection was one of the risks of GVC [3]. Additionally, the gastric drainage veins should undergo preservation or reconstruction to prevent GVC after TP, but the procedure has been reported to be difficult [2] [9] [10] [11] [12]. In our case, the gastric veins could not be preserved and we performed TG for the severe GVC and bleeding from the nasogastric tube with the strong pulsation in the LGA. Gastric veins preservation or reconstruction are recommended, but TP for cases with RCHA may require gastrectomy, including TG, due to severe GVC by increased blood flow of the LGA. As there are no guidelines for the management of GVC [3], classification of severity of GVC and indicators for gastrectomy may be needed.

Preserving the remaining stomach is better because the patient’s food intake decreases after TG. A study revealed that sub-TG was considered permissible for TP because the proximal stomach received arterial blood flow from the aortic esophageal arteries [3]. Moreover, intraoperative indocyanine green (ICG) fluorescence angiography detected a blood flow from the esophagogastric junction through the intramural capillary network in the remnant stomach during DP after the previous DG [13]. One option in TP is to assess blood flow from the esophagus to the stomach using intraoperative ICG images after clamping the LGA and consider preserving the remaining stomach. In addition, surgical techniques that preserves the gastric arteries for the preservation of stomach as much as possible should be considered.

A long-term prognosis of TP with TG is unclear because of their rarity [3] [14]. However, in recent years, good quality of life has been maintained by dietary therapy after TG and insulin and pancreatic enzyme therapy after total pancreatectomy [15] [16] [17]. In this case, the patient continued chemotherapy until recurrence for over a year while maintaining serum albumin levels by combining these treatments. Nutritional status can be maintained for a long time, even after TP with TG, by combining diet therapy, insulin, and pancreatic enzyme therapy.

## Conclusion

We experienced a case of intraoperative severe GVC requiring TG during TP with RCHA. TP with RCHA may increase the risk of severe GVC due to increased blood flow from CeA to LGA.

## Data Availability

All data generated or analyzed during this study are included in this article. Further enquiries can be directed to the corresponding author.

## Abbreviations

TP: Total pancreatectomy
GVC: Gastric venous congestion
RCHA: Replaced common hepatic artery
IPMN: Intraductal papillary mucinous neoplasia
CT: Computed tomography
SpA: Splenic artery
LGV: Left gastric vein
SMA: Superior mesenteric artery
CeA: Celiac artery
DP: Distal pancreatectomy
RGEV: Right gastroepiploic vein
GDA: Gastroduodenal artery
SSPTP: Subtotal stomach preserving TP
RGV: Right gastric vein
LGA: Left gastric artery
TG: Total gastrectomy
ICG: Indocyanine green
